# Oscillating Laser Post-Processing of NiCrCoFeCBSi/WC Thermally Sprayed Coatings

**DOI:** 10.3390/ma15228041

**Published:** 2022-11-14

**Authors:** Olegas Černašėjus, Jelena Škamat, Živilė Čepukė, Gulnara Zhetessova, Tatyana Nikonova, Olga Zharkevich, Nikolaj Višniakov, Alexandra Berg

**Affiliations:** 1 Department of Mechanics and Materials Engineering, Faculty of Mechanics, Vilnius Gediminas Technical University, 03224 Vilnius, Lithuania; 2 Laboratory of Composite Materials, Faculty of Civil Engineering, Vilnius Gediminas Technical University, 08217 Vilnius, Lithuania; 3 Department of Technological Equipment, Mechanical Engineering and Standardization, Abylkas Saginov Karaganda Technical University, Karaganda 100000, Kazakhstan; 4 Materials Research Laboratory, Vilnius Gediminas Technical University, 03224 Vilnius, Lithuania

**Keywords:** NiCrCoFeCBSi/WC coatings, oscillating laser, thermal spray, microstructure, hardness, tribology

## Abstract

In the present experimental study, the transverse oscillating laser beam technique was applied for the post-melting of metal matrix composite coatings, thermally sprayed with nickel-based self-fluxing NiCrCoFeCBSi alloy and 40 wt.% WC, to improve their hardness and wear resistance. The study was conducted using the single module optical fiber laser at 300 W power, >9554 W/cm^2^ power density, 250–1000 mm/min laser speed, 1 mm and 2 mm transverse oscillation amplitude. Scanning electron microscopy, energy dispersive spectroscopy, Knop hardness measurements, and “Ball-on-disc” dry sliding tests were conducted to study the effect of the processing parameters on the molten pool geometry and microstructure, hardness, and tribology of the processed layers. Oscillating laser processing with an amplitude of 1 mm, 250–750 mm/min laser operating speed, and sample preheating to 400 °C gave a satisfactory result: wide and shallow molten pools of ~200–350 μm in depth, hardness between ~1100 and 1200 HV0.2 and minimum cracks obtained. The coatings obtained with laser beam oscillation and preheating, and ~1150 HV0.2 hardness showed an improvement in the wear resistance and friction coefficient (~0.33) of ~2.9 times and ~20%, respectively, compared with the respective values of the coatings remelted in furnace.

## 1. Introduction

Ni-based/WC metal matrix composite (MMC) coatings are widely used for protective purposes in various fields of industry and technologies due to the excellent properties of nickel-based alloys (high strength and toughness, resistance to corrosion and wear) [1] and tungsten carbide (high melting point 2600–2800 °C, hardness 16–22 GPa and fracture toughness 28 MPa∙m^1/2^) [2], along with their very good compatibility (WC is well wetted by nickel alloy melt) [3]. Conventionally, thermal spray processes have been used for the deposition of Ni-based/WC coatings. With the rapid development of laser manufacturing in the last decades, there has been a growing interest in laser deposition and processing technologies in general, and in the laser additive manufacturing of Ni-based/WC coatings in particular. The main advantages and problems, typical for laser cladding technology of Ni-based/WC coatings, have been discussed in the previous work [4], and, as an alternative, a two-step deposition and laser post-processing technique has been considered. The technique employs a less expensive and simple-to-operate flame-spray method to form the primary composite layer, followed by the layer remelting by pulsed laser to enhance the hardness and wear resistance of the coating.

The majority of nickel-based alloys developed for thermal spray with subsequent fusing are based on the self-fluxing Ni-B-Si system, where boron and silicon act as fluxing agents, preventing oxidation and making it possible to conduct the post-melting of coatings in air. Additional alloying of self-fluxing nickel alloys with chromium (providing the solid solution hardening and the formation of carbides, borides, carboborides, or/and borocarbides), cobalt (mainly a solid solution strengthening element), iron (improving the wetting of substrate surface and participating in the hard-phase formation) and carbon (forming carbides) has enabled the development of nickel alloys with increased hardness (up to ~780 HV), and suitable for hardfacing [5]. However, the hardness of metal alloys (even plenty alloyed) is limited, and the wear resistance may be further enhanced by the addition of hard compounds, such as the most widely applied tungsten carbide. Sprayed MMC coatings with a hardness up to ~64 HRC (according to powder manufacturers) can be obtained with powder blends of high-alloyed self-fluxing nickel alloys with tungsten carbides. Ceramic materials have even greater hardness, but the brittleness typical for such materials limits their use to areas where properties other than hardness are also important, such as, for example, high-temperature oxidation resistance and low thermal conductivity in thermal barrier coatings [6]. MMC coatings, although inferior to ceramic ones in wear resistance, possess better cohesion and adhesion strength, plasticity, and impact strength, which makes them more effective in conditions of severe wear, combined with cyclic and impact loads that are typical for a wide range of different parts and components. The increase in hardness and improvement in wear resistance is achieved with an increase in the hard fraction amount; however, it leads to the loss of plasticity [7,8]. Therefore, other solutions are required to improve the performance of MMC coatings for hardfacing. One of the solutions is the addition of rare earth oxides (REOs) that enhance microhardness, wear resistance and tribological properties, provide self-lubrication properties, and reduce the friction coefficient [6,9]. It is also known that mechanical properties may be visibly enhanced by refining the microstructure to a sub-micron or nano size that may be reached by spraying nano-sized materials or by the application of concentrated energy sources such as electron or laser beams [10]. Many studies in the field show that laser melting of metals and MMC materials forms a very fine microstructure, and improves their metallurgical, mechanical, and other properties including hardness. With the development of laser equipment, new processing techniques have become available to overcome some disadvantages typical for laser melting of material surfaces.

The laser-remelted surface layer is formed in a pass-by-pass manner, with a previous pass overlapped by a subsequent pass and with a “saw-shaped” profile in its cross-section with geometry predetermined by the non-uniform power distribution, which typically can be assumed approximately to be Gaussian, within the laser beam [11]. To overcome the non-uniform power distribution and to produce the desirable energy distribution over a defined surface area, a beam oscillating technique was developed—firstly for the electron beam [12] and, later, for the laser [13]. Recently, laser oscillating welding has gained growing interest, mainly in the field of welding of dissimilar metals [14] and aluminum alloys [11,15,16,17,18], and also for other alloys such as low carbon steel [19], stainless steel [20,21], and Invar alloy [22]. During oscillating laser processing, the laser head moves along the defined path at a constant velocity, while the laser beam is oscillated at high frequency by a certain trajectory, for example, sinusoidal, square, triangular, circular, elliptical, etc. As a result, the power distribution within a single pass is changed, counteracting the fast quenching of the melt pool and reducing the severe thermal gradients [13]; the melt pool becomes wider and shallower, the bottom of the melt pool is flat [17]; the width of the melt pool is predetermined by the oscillation amplitude [23]. It is pointed out [23] that the oscillating technique enable researchers to stabilize the laser-welding process and to improve the process efficiency due to increased volume of the melt pool. Ai et al. in their study [17] observed a decrease in the molten pool maximum temperature and flow velocity with an increase in oscillation amplitude or frequency; this resulted in a more stable and shallower melt pool and the reduction of aluminum alloy weld porosity. The melt pool stirring effect leads to the improvement of metallurgical behavior of the weld associated with the deflection of oriented dendrites growth and, as a result, the transition to equiaxed grain microstructure and grain refinement [15,22]. According to finite element (FE) analysis results [22], the oscillation provides a more uniform temperature distribution in a molten pool center. Moreover, FE simulation shows that oscillating laser welding can relieve the stress concentration effects in weldments. It is reported in [20] that the increase in oscillation amplitude and frequency reduces as-weld residual stress and distortion. Kim et al. [18] in their study found that solidification cracks in aluminum alloy welds disappeared at 5 Hz frequency laser weaving.

The majority of available studies on the oscillating laser technique are in the field of welding technologies. However, the reported results make this technique promising also for other manufacturing fields where laser beam energy is used to fuse materials and obtain the solidified microstructure from the liquid state. Thus, Xia et al. [24] used the oscillating technique for laser melting deposition of nickel-based superalloy reinforced by WC. Authors pointed out that circular oscillating provided pore-free and crack-free layers with a combined equiaxed-columnar microstructure and uniformly distributed reinforcing carbide phase. As a result, sound composite coatings with enhanced hardness and improved wear resistance were obtained.

In the present study, the oscillating laser melting technique was applied to post-process the deposited nickel-based/WC coating. In the previous work [4], the inexpensive and simple-to-operate flame spray method enabled us to obtain composite coatings of stable thickness and uniform distribution of micro-sized WC particles, while laser post-melting led to the formation of ultrafine microstructure consisting of submicro-sized and nano-sized W-rich dendrites in an Ni-based matrix with microhardness of ~1100 HK0.2 and wear resistance up to ~2.4 times better in comparison with as-sprayed coating. The sensitivity of the coatings to crack formation was reduced with the reduction of laser power density. However, the problem was not fully resolved. For the further development of this two-step coating technology, the effects obtainable with oscillating laser processing, such as a wider and shallower molten pool, reduced thermal gradient, improvement of metallurgical behavior and reduction of residual stresses, are of high scientific and applied interest. To the best of the authors’ knowledge, the oscillating laser processing has not yet been studied as a post-melting technique for the MMC coatings thermally sprayed from the powder mixture of self-fluxing nickel alloy and tungsten carbide. The effect of melt pool stirring, change of melt flow direction and heating/cooling conditions appearing with laser beam oscillation on the cracking and hardness of the coating determining the wear resistance properties, has not been not investigated earlier. The trend of molten pool geometry change with oscillation amplitude is predictable; however, the absolute values of dimensions are not known. To study the possible effects, the experimental study was conducted, during which the thermally sprayed Ni alloy/WC coatings were laser-remelted by applying different oscillating amplitudes and combining oscillating laser processing with preheating of the coatings. The previous study has shown that partial melting of substrate is needed during laser processing to improve the coating adhesion, which is typically not satisfactory after the flame spray. However, the melting of substrate causes the admixing of the substrate alloy melt into the coating molten pool, and a change in its elemental and phase composition, and that may deteriorate the laser-processed coating properties, including corrosion and wear resistance. Therefore, at this stage of the study, the thermally sprayed coatings were firstly heat-treated in an electric furnace to obtain the partial melting of the coating, and the formation of a metallurgical bond between the deposit and the substrate. The experimentally obtained coatings were then investigated to determine the molten pool geometry, the formation of cracks, hardness distribution across the coatings, and their tribology.

## 2. Materials and Methods

### 2.1. Coatings Materials and Deposition

For the oscillating laser remelting experiments, thermally sprayed coatings were deposited using a powder mixture (fraction 38–125 μm) consisting of self-fluxing Ni-based alloy (composition in wt.%: C—0.4; Cr—13.8; Fe—3.9; Co—11.8; B+Si—7.9; Ni—balance) and 40 wt.% of WC. For depositing, an oxy-fuel flame spraying torch Rototec 80 (Castolin Eutectic, Lausanne, Switzerland) and a manipulator Motoman 100 (Yaskawa Nordic, Torsås, Sweden) were employed with the following parameters: spraying distance 170 mm; torch operating speed 250 mm/s; a space between adjacent passes 5 mm; neutral oxygen–acetylene flame. The substrate was flame preheated to a temperature of ~230–250 °C; the final coating thickness of ~1.2–1.3 mm was obtained by eight sprayed layers. The coating was deposited on structural steel S235 plates having dimensions 150 mm × 40 mm × 8 mm. The surface of the plates was cleaned with isopropyl alcohol, grit-blasted, again washed with isopropyl alcohol, and dried in hot air before spraying. To improve the coating adhesion with the substrate and to compact the deposited layer, the as-sprayed samples were preheated in the electric furnace at 1300 °C for 2.5 min. and afterwards cooled in air at room temperature.

### 2.2. Laser Post-Processing Details

**Figure 1:**
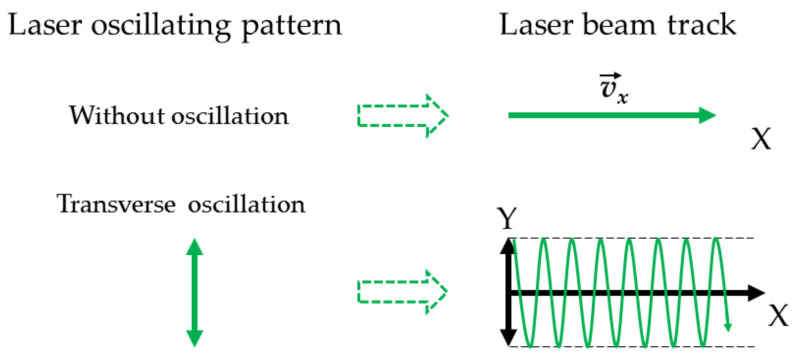
Schematic diagrams of beam oscillating pattern and the resultant track of laser beam.

The remelting experiments of the deposited coatings were performed using a fiber laser machine FANUCI^®^ PRO 1500 (Fanuci, Gdansk, Poland) with a single module optical fiber laser source and oscillation function; wavelength—1070 nm. The main processing parameters were as follows: continuous laser emitting; laser power—300 W; laser spot diameter—1 mm; laser operating speed between 250 and 1000 mm/min; transverse oscillation at a frequency of 110 Hz; oscillation amplitudes—1 mm and 2 mm (the distance between the laser beam center axis); shielding gas—argon (consumption—15 L/min). The efficiency of the laser was >25%, that is, the average laser beam power density was >9554 W/cm^2^ during the experiments, providing the processing in the melting mode. The laser remelting experiments were conducted by applying the parameters listed in Table 1 with and without oscillation (Figure 1), and with and without sample preheating to a temperature of 400 °C.

### 2.3. Analysis Methods

For the microscopical analysis of the coatings, a scanning electron microscope SEM JEOL JSM-7600F (JEOL, Akishima, Japan) equipped with an energy dispersive spectrometer IncaEnergy 350 (Oxford Instruments, Oxford, UK) for X-ray microanalysis was used. The main analysis parameters were: 10 kV accelerating voltage; ~8 mm working distance; mixed secondary and backscattered electron signal for imaging. For the microscopic analysis, the samples were sectioned, mounted, grounded, and polished using conventional techniques for metallographic analysis (the last polishing step was performed using 0.2 μm fumed silica suspension). The geometric parameters (width and depth) of the molten pool of individual laser-processed passes were determined using SEM images made at ×100 magnification for non-oscillated molten pools and ×50 magnification for the pools obtained with oscillation.

The hardness study was conducted using the microhardness tester Zwick Roell ZHμ (ZwickRoell GmbH & Co. KG, Ulm, Germany). Measurements were carried out using the Vickers method with 200 g load and 15 s duration on the mounted, ground, and polished cross-sections of the samples. The average values of microhardness were calculated as an arithmetic mean of individual measurements made across the molten pool width (Av_horiz.) and depth (Av_vertic.), and presented in the paper along with the average microhardness (Av) calculated as a mean of *Av_horiz.* and *Av_vertic.* The paper presents the microhardness distribution profiles across the width of the coatings obtained by making indentations at the depth of 50 μm from the surface with the step of 100 μm.

The “Ball-on-disc” dry sliding tests were conducted to evaluate the tribological properties of the experimental coatings. The tests were performed using a Microtest tribometer. Before the test, the surface of the sample to be tested was pre-polished to Ra ~0.46 µm. The tribological testing parameters were as follows: sliding distance—200 m; sliding speed—200 rpm; radius of the trajectory—0.7 mm; load—20 N; temperature of the experiment—22 ± 1 °C; indenter—6 mm diameter ball made of tempered stainless steel AISI52100. The profilometer TR-200 with an accuracy ±0.01 μm was employed to measure the micro-roughness of the tested surfaces. The wear resistance of the coatings was evaluated by mass loss. The analytical balance Precisa XR 205SMDR with an accuracy of 0.00001 g was used to measure the mass of the samples before and after the tribology test. Before weighing, the test samples were cleaned in an ultrasonic bath for 15 min. The wear rate of the coatings was calculated by dividing the mass loss by the sliding distance and expressed in μg/m. The coating wear resistance was calculated by dividing the sliding distance by the mass loss and expressed in m/μg. The average values of three tests are presented in the paper. The friction coefficients were calculated after the test data for the first 20 m of the sliding distance were eliminated; the average values are presented in the paper with standard deviation.

## 3. Results and Discussion

### 3.1. Characterization of the Coating Remelted in Furnace

**Figure 2:**
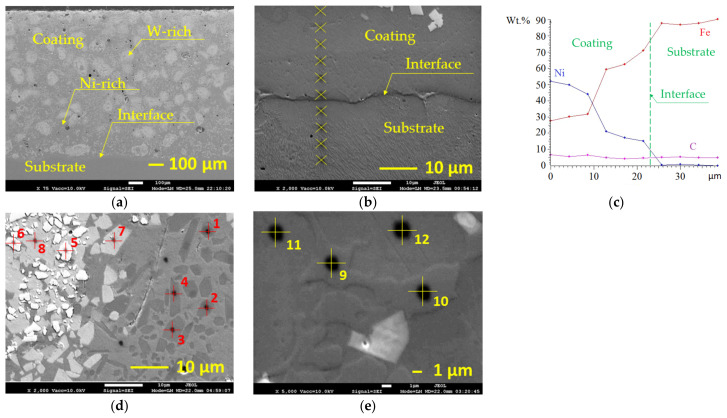
SEM micrographs of the coating remelted in a furnace (**a**,**b**,**d**,**e**) and the graph of elemental distribution across the interface between the coating and the substrate (**c**): (**a**) general view of the remelted coating; (**b**) enlarged view of interface zone with marked points of EDS analysis; (**d**) microstructure of the coating representing the presence of different types of hard precipitates with marked points of EDS analysis; (**e**) enlarged view of metal matrix microstructure with marked points of EDS analysis.

As previously noted [4], the as-sprayed coating has discontinuous microstructure with a significant number of voids and gaps between splats, a wide sharp interface between the deposited layer and the substrate and, subsequently, poor cohesive and adhesive strength. In the present study, the laser processing experiments were performed on the coatings thermally sprayed and remelted in a furnace (Figure 2a). After the remelting process, a monolithic layer with visibly reduced porosity (from ~19.2% in as-sprayed coating to ~3.6% in furnace remelted coating) was formed; the agglomerated tungsten carbide particles were partially dissolved in the matrix of nickel alloy. Due to the partial melting of nickel-based phases, the coating layer wetted the substrate surface and formed a uniform interface (Figure 2b). The distribution of elements across the obtained interface showed an increased concentration of iron in the coating near the interface, indicating that iron diffusion took place and a strong metallurgical bond was formed (Figure 2c). The typical microstructure of the as-sprayed and remelted in furnace coating, exhibiting a variety of the phases formed, is shown in Figure 2d. The obtained microstructure consists of at least three types of hard precipitations distributed in a two-phase metal matrix consisting of γ-Ni solid solution and Ni-B eutectic (Figure 2e). According to EDS analysis (Table 2), besides nickel, the solid solution phase has iron, chromium, cobalt, and silicon dissolved in γ-nickel. The hard precipitations observed are non-fully dissolved WC particles, chromium-rich carbides (or, possibly, carbo-borides), with a low number of nickel (~4 at.%) and iron (~2 at.%) atoms replacing chromium, and complex carbides rich in nickel (~20 at.%) and chromium (~15 at.%) also containing tungsten (~8 at.%), silicon (~4.5 at.%), and cobalt (~2.5 at.%). Taking into account that EDS typically gives carbon and boron concentrations higher than they really are, the ratios of C+B atoms to metal atoms in the observed carbides are lower than can be calculated from Table 2. Correspondingly, the carbides of type Cr_3_C_2_ and/or Cr_7_C_3_ (where Cr atoms are partially replaced by Ni, Fe, Co, and W) may be assumed along with M_2_C or M_7_C_3_ type carbides, where M is mainly Ni, Cr, and W.

### 3.2. Laser-Remelted Coatings: The Effect of Oscillating Amplitude, Laser Operating Speed and Preheating Temperature on the Molten Pool Geometry

**Figure 3:**
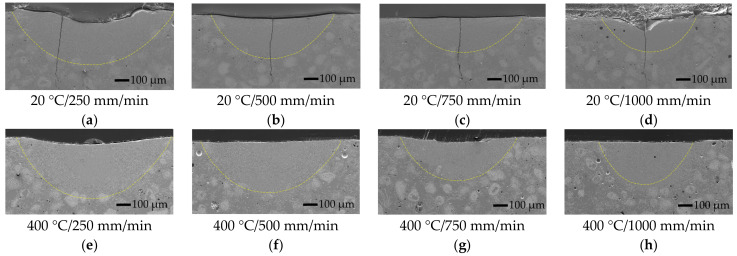
Cross-sectional SEM micrographs of non-oscillated molten pools obtained without (**a**–**d**) and with (**e**–**h**) preheating and a varying laser operating speed between 250 and 1000 mm/min.

**Figure 4:**
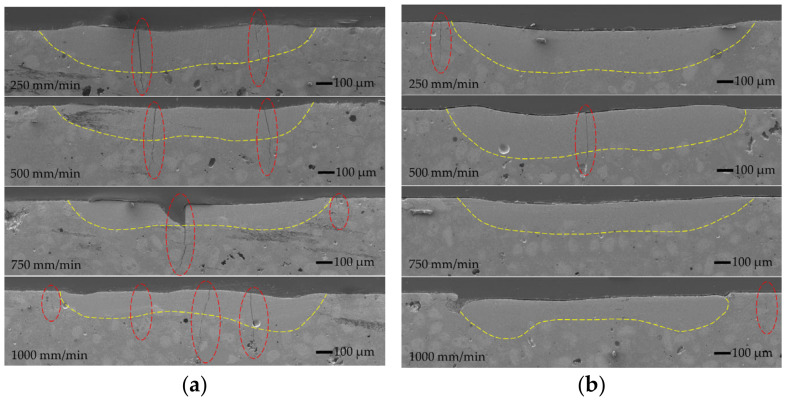
Cross-sectional SEM micrographs of molten pools oscillated with 1 mm amplitude without (**a**) and with (**b**) preheating and a varying laser operating speed between 250 and 1000 mm/min: red dotted ovals show the cracks.

**Figure 5:**
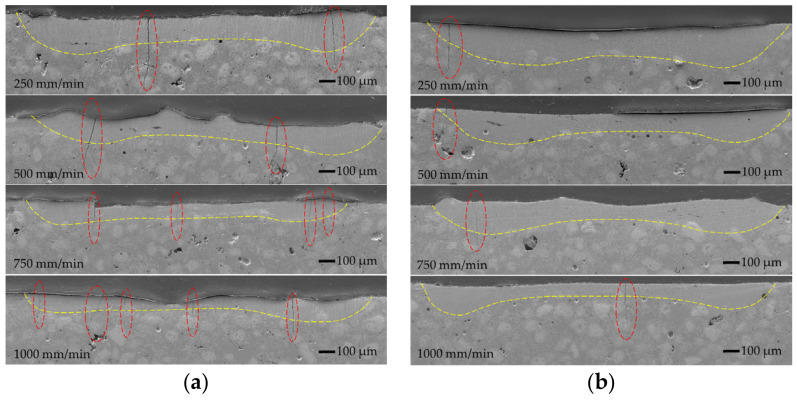
Cross-sectional SEM micrographs of molten pools oscillated with 2 mm amplitude without (**a**) and with (**b**) preheating and a varying laser operating speed between 250 and 1000 mm/min: red dotted ovals show the cracks.

The intermediate stage of furnace remelting of the coating created a good metallurgical bond between the coating and the substrate before the laser processing. In addition, there was no need to fuse the substrate to obtain acceptable adhesion during the laser remelting process. All the laser-melt pools formed with and without oscillation were obtained in the coating layer without the substrate melting (Figure 3, Figure 4 and Figure 5).

The general view of the molten pools formed without oscillation, and with and without pre-heating is presented in Figure 3.

**Figure 6:**
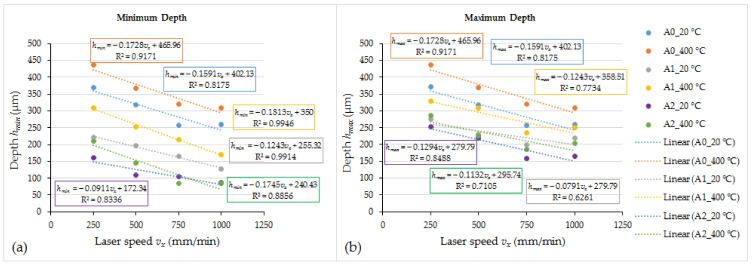
The relationship between the minimum (**a**) and the maximum (**b**) depth of molten pools and laser operating speed, applied oscillation amplitude and preheating temperature; for non-oscillated molten pools *h_min_* = *h_max_*; oscillation amplitude A0—0 mm (no oscillation); oscillation amplitude A1—1 mm; oscillation amplitude A2—2 mm.

**Figure 7:**
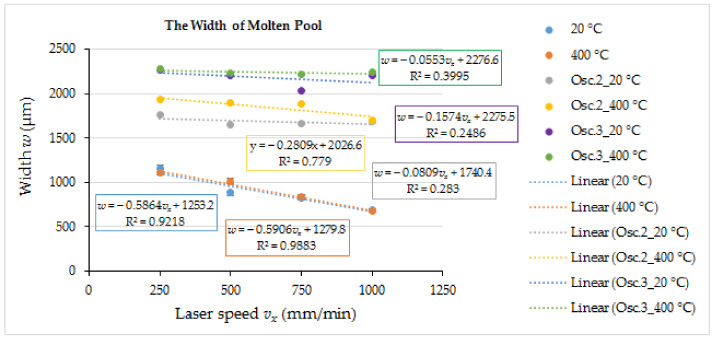
The relationship between the width of molten pool (measured in the widest part of the pool) and laser operating speed, applied oscillation amplitude and preheating temperature; oscillation amplitude A0—0 mm (no oscillation); oscillation amplitude A1—1 mm; oscillation amplitude A2—2 mm.

The laser melting of the coating without oscillation and preheating at the lowest laser speed of 250 mm/min providing the highest heat input formed the molten pool of ~1155 μm wide and ~370 μm deep (Figure 6 and Figure 7). The increase in the laser operating speed up to 1000 mm/min resulted in a gradual reduction of the molten pool width and depth to ~685 μm and ~260 μm, respectively, which is by ~41% and ~30%, respectively. With the introduction of transverse oscillation at the amplitude equal to laser spot diameter (1 mm) and to double laser spot diameter (2 mm), the molten pool width increased ~1.5–2.5 and 2–3.2 times, respectively, while the maximum depth of the molten pool reduced (due to power density dissipation) by ~16–27% and ~31–39%, respectively, depending on the laser speed. The thickness of the molten pool center reduced even greater—~3 times. The geometry of molten pools also showed that a more uniform distribution of power density across the molten pool’s cross-section (Figure 4) was achieved with the oscillation with the amplitude of 1 mm, while a further increase in the amplitude led to the concentration of power in the molten pool edges and the formation of the non-uniform bottom of the melt pool (Figure 5). The preheating of the samples before laser processing to a temperature of 400 °C did not have a significant influence on the width of the molten pools, but resulted in an increase of the molten pool depth by up to ~40%.

All the laser-processed samples without preheating showed high sensitivity to the formation of cracks. There are three main reasons for crack formation in the layers obtained by the laser process: high thermal stresses due to high heating and cooling rates [25]; the presence of hard brittle phases, such as carbides and borides [26]; and the presence of low-melting-point phases, such as Ni-B eutectic. It is well known from the welding theory that local (non-uniform) heating causes the formation of residual tensile stresses (thermal stresses) that increase with cooling and solidification. When the level of residual stresses is high, the brittle phases cannot withstand the tension due to limited plasticity; if residual stresses reach a high level while the low-melting-point eutectic phases are not yet solidified, the melt will not withstand the tension and will crack. In the NiCrCoFeCBSi/WC systems, the presence of hard and eutectic phases is inevitable. Therefore, the reduction of thermal stresses is the main way to reduce the crack formation in laser-processed layers. The results of the microscopic observation showed that oscillation had not improved the conditions; on the contrary, the number of cracks increased significantly with the increasing width of the molten pool. As noted by other authors [27,28,29,30,31,32], in preheated samples the temperature gradient between the molten pool and the colder part of the sample, which restrains the free shrinkage of the molten pool during its solidification, reduces and, consequently, thermal stresses reduce as well, resulting in fewer or no cracks in the laser-cladded layers. The preheating temperatures applied in the works mentioned above varied between 200 °C and 1173 °C. The reported results also showed that preheating could lead to a significant reduction of hardness. Taking this into account, a moderate preheating temperature of 400 °C was chosen in the present study. The visible reduction in crack formation was reached with the samples preheated to 400 °C; crack-free layers were obtained without oscillation and the layers made with oscillating laser melting had a significantly lower number of cracks.

### 3.3. Laser-Remelted Coatings: The Effect of Oscillating Amplitude, Laser Operating Speed and Preheating Temperature on the Microstructure of the Processed Layers

**Figure 8:**
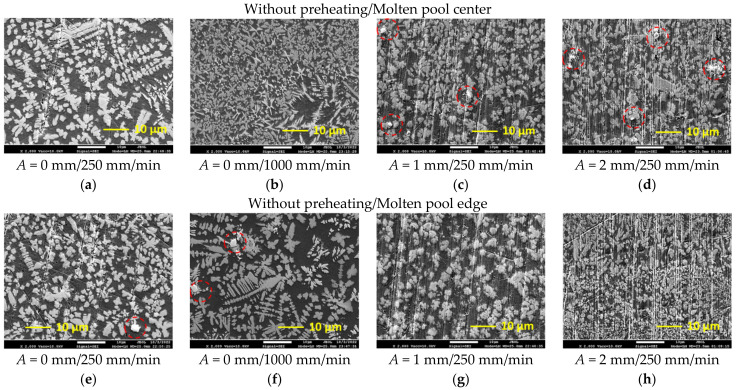
Microstructure of the central (**a**–**d**) and edge parts (**e**–**h**) of the molten pools obtained by laser remelting without preheating: (**a**,**b**,**e**,**f**)—without oscillation; (**c**,**g**)—with 1 mm oscillation amplitude; (**d**,**h**)—with 2 mm oscillation amplitude; the non-melted WC particles are marked with red circles.

**Figure 9:**
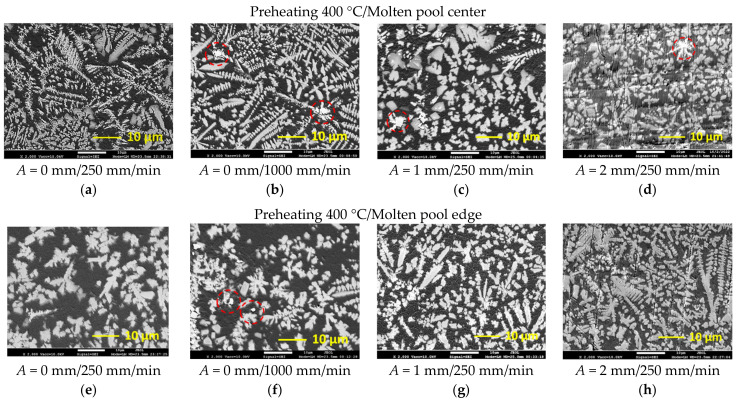
Microstructure of the central (**a**–**d**) and edge parts (**e**–**h**) of the molten pools obtained by laser remelting with pre-heating at 400 °C: (**a**,**b**,**e**,**f**)—without oscillation; (**c**,**g**)—with 1 mm oscillation amplitude; (**d**,**h**)—with 2 mm oscillation amplitude; the non-melted WC particles are marked with red circles.

The increased hardness of laser-processed layers is mainly predetermined by very rapid cooling and solidification processes that typically form an ultra-fine submicro- and/or nano-sized microstructure. Laser processing parameters applied in the present study also provided a very fine microstructure (Figure 8 and Figure 9). The temperature gradient *G* and the growth rate *R* at the solidification front determine the solidification microstructure. The product *GR* (that in fact is the cooling rate) determines the size of the microstructure and the ratio *G/R* determines its morphology. The higher the cooling rate *GR*, that is, the shorter the solidification time, the finer is the structure formed. With the reduction of *G/R,* the transition to equiaxed microstructure is observed.

For the laser surface melting process, high *GR* and *G/R* are generally typical. Therefore, all laser-processed samples in the present study showed a very fine microstructure. However, the non-uniform distribution of power density in the laser beam provides uneven heating, and the variation of laser operation speed changes the cooling rate. With the introduction of transverse oscillation of the laser beam, the velocity of molten pool increases due to additional lateral movement. The application of the sample preheating reduces *G* and *R*; consequently, the *GR* should be decreased while the *G/R* ratio is determined by the specific *G* and *R* values. As a result, the difference in the obtained microstructure morphology and size may be observed for the samples processed at different conditions here.

Figure 8a,e show the fine equiaxed dendritic microstructure of solidified molten pool obtained at 250 mm/min laser speed providing the highest heat input. At the longer heating and lower cooling rate, provided by a laser speed of 250 mm/min, a very similar microstructure in the center and edge parts of the molten pool was obtained; however, slightly shorter and thinner dendrites can be observed (Figure 8e) near the molten pool edge. In addition, the presence of non-fully dissolved WC particles was established there, which may be related to the lower temperature of the melt and faster cool down below the temperature at which the dissolution of WC might take place. The increase in the laser operating speed by four times (up to 1000 mm/min) led to the *GR* rise and resulted in a significant microstructure refinement in the center of the molten pool (Figure 8b). At this laser speed, the difference in heating and solidification conditions between the center and edge parts of the molten pool is more visible (Figure 8b,f).

With the introduction of transverse oscillation of the laser beam, the molten pool gains additional lateral movement at a certain velocity in addition to its main velocity along the processing pass. As a result, the sum velocity of the oscillated molten pool is greater than that of the non-oscillated pool, that is, *R* should be increased. The re-distribution of power density across the molten pool cross-section results in a decrease of melt temperature, which is confirmed by the non-fully dissolved WC particles as well (Figure 8c,d). Since the temperature difference decreased, the *G* should be decreased. As a result, the size of microstructural components in the center part of the molten pool did not change visibly, as compared with that obtained without oscillation. However, no long dendrites were observed and mostly short and equiaxed dendrites were formed, which might be related to the stirring effect due to oscillation, resulting in a dendrite fragmentation. It should be also pointed out that with the oscillation amplitude of 1 mm, a more uniform microstructure across the molten pool was obtained, while the increase of the amplitude up to 2 mm resulted in an increase of microstructure unevenness.

The preheating of the samples before laser processing to 400 °C decreased the temperature gradient *G*. Correspondingly, both the *G/R* and *GR* decreased and transition to coarser and more developed dendritic morphology was observed in preheated laser-processed samples.

**Figure 10:**
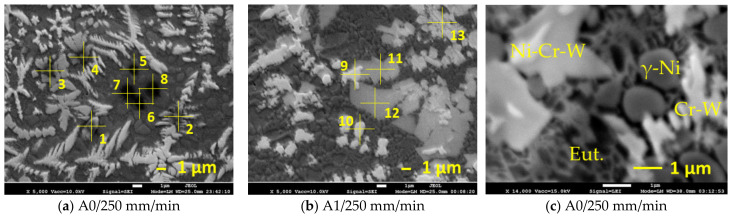
SEM images of the microstructure obtained without oscillation (**a**) and with oscillation amplitude *A* = 1 mm (**b**) at 250 mm/min laser speed; 1–13—points of EDS analysis (Table 3); (**c**)—enlarged view of microstructure after etching showing four main constituents of the structure: γ-Ni—grains of nickel-based solid solution; Eut.—eutectic; Ni-Cr-W—Ni,Cr,W-rich carbides; Cr-W—Cr,W-rich carbides.

EDS microanalysis showed that all the samples obtained with laser processing had similar phase constituents (Figure 10 and Table 3). The metal matrix consisted of γ-Ni solid solution, with Co, Cr, Fe, Si, W dissolved in it, and Ni-B-Si based eutectic phase, which also contained some Co, Cr and Fe. Two types of hard phases were identified: Cr, W-rich carbides with higher carbon concentration (which look almost white in SEM images (Figure 10a,b, points 1, 2, 9, 10) and Ni,Cr,W-rich carbides with lower carbon concentration and also containing Co, Fe, and Si (which look light grey in SEM images (Figure 10a,b, points 3, 4, 11, 12). In addition, in point 13, the ratio of metallic components differed from that in points 3, 4, 11 and 12, indicating that one more type of carbide might be present in the structure. It should be also pointed out that residuals of non-fully dissolved WC particles were observed in the laser-remelted layers. The enlarged view of the microstructure obtained after laser processing showed four main visible constituents—grains of γ-Ni solid solution (Figure 10c, “γ-Ni”), areas having typical eutectic morphology (Figure 10c, “Eut.”), and at least two types of hard precipitates, most likely carbides (Figure 10c, “Ni-Cr-W” and “Cr-W”).

**Figure 11:**
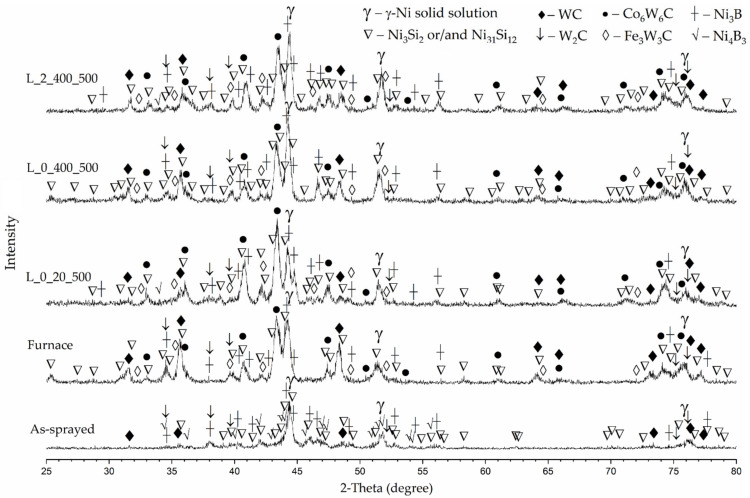
XRD patterns of the as-sprayed coating, coating remelted in furnace and laser post-processed coatings (L_0_20_500; L_0_400_500; L_2_400_500); L_0_20_500—laser processing without oscillation and preheating at a laser speed 500 mm/min; L_0_400_500—laser processing without oscillation with preheating to 400 °C at a laser speed 500 mm/min; L_2_400_500—laser processing with oscillation amplitude A = 2 mm with preheating to 400 °C at a laser speed 500 mm/min.

According to X-ray diffraction analysis results (Figure 11), the main reflections observed in the XRD pattern of the as-sprayed coating are attributable to a γ-Ni solid solution phase having a face-centered cubic (fcc) lattice with parameter a = 3.545 Å and tungsten carbide phase (WC). Besides, minor reflections of much lower intensity were observed; with high probability these reflections might be attributed to W_2_C carbide and compounds typical for complex Ni-B-Si eutectic, such as Ni_3_B, Ni_4_B_3_, Ni_3_Si_2_ and Ni_31_Si_12_, which was consistent with the phase composition of the powder mixture used for spraying [33]. It should be noted that the peaks observed in the XRD pattern of the as-sprayed coating had a lower intensity compared with those in the remelted coating, which might be related with significant porosity of the coating in the as-sprayed state. The melting of low-melting-point eutectic phases, partial dissolution of WC particles in nickel-based matrix and admixing of Co-based binder of WC to the Ni-based molten pool took place during the remelting process in the furnace. The solidification ran at a lower rate compared with that during the thermal spray and atomization process, by which the Ni-based powder was manufactured. As a result, the changes in phase composition occurred. Additional intensive reflections attributable to Co_6_W_6_C type carbide were observed. According to [34], they are typical for WC-Co systems and may be related to the increased concentration of cobalt in the molten pool due to the dissolution of Co-based binder of WC particles. Moreover, low-intensive reflections were observed that might be attributed to Fe_3_W_3_C type carbide. The Ni_4_B_3_ phase most likely disappeared and did not form after the remelting, while the peaks typical for the Ni_3_B phase were observed. SEM/EDS analysis revealed the presence of chromium carbides in the coatings remelted in furnace. However, no reflections that might be attributed to these compounds were observed, which can be explained by their low amount, which was not enough to be determined by XRD analysis. The XRD patterns of laser-remelted coatings did not differ significantly from those of furnace-remelted coatings. However, the visible intensity reduction of the main reflections (2-theta angles ~35.9°, ~48.7° and ~73.4°) attributable to the WC phase should be noted. It can be explained by significant dissolution of WC particles, as the microscopic analysis showed only very few residuals of WC in laser-remelted coatings. Based on the results of XRD, SEM and EDS analyses, it can be assumed that the hard precipitations formed in the coatings after laser remelting are represented mainly by M_12_C type carbides and, to a lesser degree, by M_6_C and M_2_C carbides along with WC residues.

### 3.4. Laser-Remelted Coatings: The Effect of Oscillating Amplitude, Laser Operating Speed and Preheating Temperature on the Hardness of the Processed Layers

**Figure 12:**
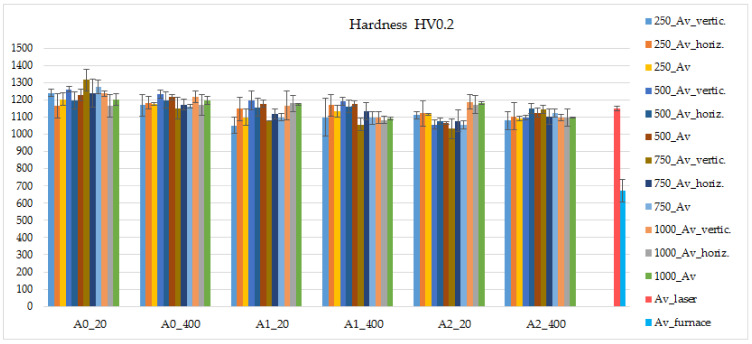
Average hardness HV0.2 of the coating remelted at different parameters: Av_horiz.—average hardness calculated from the horizontal hardness distribution values; Av_vertic.—average hardness calculated from the vertical (depth) hardness distribution values; Av—the mean of Av_horiz. And Av_vertic.; Av_laser—the mean of all Av values; 250, 500, 750, 1000—indicates laser speed; 20 and 400—preheating temperature; A0, A1, and A2—0 mm, 1 mm, and 2 mm oscillation amplitude.

**Figure 13:**
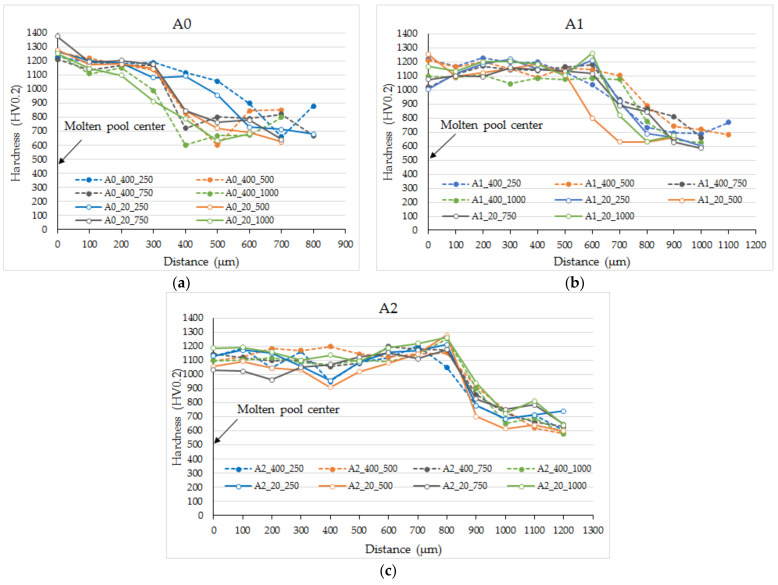
Hardness HV0.2 distribution across molten pool width: 250, 500, 750, 1000—indicates laser speed; 20 and 400—preheating temperature; A0, A1, and A2—0 mm, 1 mm, and 2 mm oscillation amplitude.

The results of the hardness study are presented in Figure 12 and Figure 13. The hardness of the as-sprayed coating was not evaluated due to very poor cohesion and elevated porosity. The furnace-remelted coatings remelted showed ~670 HV0.2 hardness (Figure 12: Av_furnace) while individual values varied in a wide range from 530 to 810 HV0.2, which is related to non-uniform distribution of hard phases in the metal matrix (at the micro scale) and their big size compared with indentation impression. With the laser remelting, the hardness of coatings was significantly improved: the average hardness of laser-processed coatings ranged from ~1030 to ~1310 HV0.2, which is 54–96% higher than that of the coatings remelted in furnace. Non-oscillated coatings processed without preheating showed the greatest hardness (1170–1310 HV0.2). The preheating resulted in insignificant hardness reduction (1150–1230 HV0.2). With the introduction of oscillation, some additional hardness decrease was determined that cannot be explained by morphological and size difference only. Taking into account the spread of values, the absence of pronounced dependencies of hardness on the parameters, and the reduction of the molten pool depth with oscillation, it may be suggested that the concentration of WC particles in the surface layer to be laser remelted may dominate the final hardness here. The distribution of the WC in furnace-remelted coating was found to be uniform at the sub-macroscopic scale, while at the microscopic scale it was not so even. With the increase of the molten pool volume, the effect of WC distribution becomes less expressed, while at a smaller/shallower molten pool it may result in a hardness drop or leap. It is to be believed that some increase of the laser power at the oscillating laser processing mode would produce a deeper molten pool with a bigger volume more stable hardness.

The hardness distribution was investigated in the direction of the molten pool depth and width. As can be seen from Figure 13, for the non-oscillated molten pool, the hardness was greatest in the middle and dropped near the molten pool edges (Figure 13a). Molten pools obtained with laser oscillation showed a more uniform hardness distribution with a slight hardness leap at the edges, less expressed at 1 mm amplitude and more expressed at 2 mm (Figure 13b,c), which correlates with power distribution indicated by molten pool geometry. At oscillation amplitude 2 mm, a more visible local drop in hardness (900–950 HV0.2) might be observed, which might be related to the too-small molten pool depth and a more expressed effect of WC distribution (as was discussed above).

In the context of using laser beam oscillation and obtaining a wider and shallower molten pool, also taking into account the results of geometry, crack formation, and hardness tests, it can be noted that processing with an amplitude of 1 mm, 250–750 mm/min laser operating speed and preheating gave a satisfactory result: wide and shallow molten pools of ~200–350 μm in depth, hardness between ~1100 and 1200 HV0.2, and minimum cracks obtained.

### 3.5. Tribology of the Coatings

**Figure 14:**
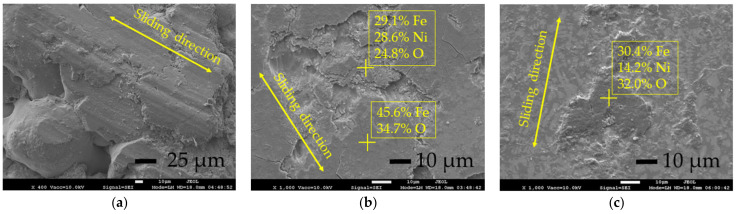
SEM images of wear tracks after two body dry sliding test of as-sprayed (**a**), sprayed and remelted in furnace (**b**) and laser processed (**c**) coatings: the concentrations of elements in marked points are given in wt.% (by EDS).

The tribological study under dry conditions was performed for three sample series—the as-sprayed coatings, furnace-remelted coatings, and a laser-remelted coating. To provide enough surface area for the sliding test, the coatings for laser remelting were chosen from those obtained at 2 mm oscillation. The samples from the series processed with preheating at 500 mm/min laser speed and showing the greatest average hardness (~1150 HV0.2) were tested. The results are presented in Table 4. The view of the wear tracks after two-body dry sliding tests is shown in Figure 14. As can be observed from Figure 13a, plentiful delamination of surface layers of the as-sprayed coating, predetermined by the poor coating cohesion, took place during the loading, and subsequently resulted in a significant mass loss and increased friction coefficient. After the remelting in a furnace, the cohesion of the coating and its hardness were improved and a monolithic layer was obtained. Consequently, the coating mass loss and the friction coefficient reduced drastically by ~26 times and by ~22%, respectively, and the wear pattern changed. It can be observed from Figure 14b that the surface showed the signs of brittle crumbling (associated with the non-uniform distribution of hard phases and occurring in the zones of their accumulations) and a smooth thin layer showed the presence of cracks and delamination. According to EDS analysis, this layer consisted of 45.6 wt.% Fe and 34.7 wt.% O, which is iron oxide. The wear debris present on the surface showed increased Fe and O concentrations as well. Thus, it can be assumed that mainly the wear products of the contra-body (steel ball) accumulated on the surface of the coating during the sliding test, which then were repeatedly plastically deformed, formed a brittle layer, and finally delaminated. After the delamination of that layer, the uneven surface of the coating was exposed. The additional remelting of the coating with laser formed an ultra-fine microstructure and produced a hardness increase of ~72%. As a result, the coating wear resistance additionally improved by ~2.9 times. The surface of the wear track was very smooth, with the presence of some wear debris mainly consisting of iron oxides; no signs of brittle crumbling were observed, and what was related to the uniform distribution of hard precipitation well incorporated in the metal matrix (Figure 14c). The wear process was found to be very even, without the loss of the coarse bits of the coating; the same was also confirmed by reduced mass loss and friction coefficient.

## 4. Summary

In the present study, the flame sprayed NiCrCoFeCSiB/WC coatings were post-melted using a fiber laser FANUCI^®^ PRO 1500 machine with the single module optical fiber laser source and oscillation function. The effect of the laser operating speed, oscillation amplitude, and preheating on the molten pool geometry, cracks formation, microstructure, and hardness of the coatings was investigated. The tribological behavior of the laser-remelted coatings with oscillation was compared with the as-sprayed coating and the coatings sprayed and remelted in furnace. The results of the experimental research presented in this paper are summarized below:

- The remelting of the as-sprayed coatings in an electrical furnace at 1300 °C for 2.5 min provided a near-monolithic layer (of ~670 HV0.2 hardness) with low residual porosity (~3.6%), improved coating cohesion, and a strong metallurgical bond between the coating and the substrate. This enabled us to conduct the laser remelting experiments without melting the substrate surface and avoiding substrate material admixing to the molten pool.
- The operation of the optical fiber laser at the continuous emitting mode with a laser power of 300 W and laser speed between 250 and 1000 mm/min provided processing of flame-sprayed and remelted in furnace NiCrCoFeCSiB/WC coatings in a melting mode. The width and the depth of the non-oscillated molten pools depended on the laser speed, and were in the ranges of 1155–685 μm and 370–260 μm, respectively. With the introduction of transverse oscillation at the amplitude equal to the laser spot diameter (1 mm) and to double laser spot diameter (2 mm), the molten pool width increased ~1.5–2.5 and 2–3.2 times, respectively, while the maximum depth of molten pool (due to power density dissipation) reduced by ~16–27% and ~31–39%, respectively, depending on the laser speed. The thickness of the molten pool center (in its thinnest place) reduced even greater—up to ~3 times. The preheating of the samples to a temperature of 400 °C before laser processing did not influence significantly the molten pool width, but resulted in an increase of the molten pool depth by up to ~40%.
- Laser processing at 9554 W/cm^2^ power density and 250–1000 mm/min laser speed without preheating formed cracks in all the specimens obtained; laser beam oscillation increased the crack appearance. Preheating to 400 °C prevented crack formation in non-oscillated samples and visibly reduced it in oscillation mode.
- The combination of laser oscillation at the amplitude equal to laser spot diameter (1 mm) at 250–750 mm/min laser speed and preheating to 400 °C produced a wide shallow molten pool with quite a flat bottom and uniform microstructure and hardness distribution, and reduced the appearance of cracks. Taking into account the results of the study, it may be summarizes that it is not reasonable to apply an oscillation amplitude greater than the laser spot diameter and high laser operating rates.
- The microstructure morphology and size of the layers processed at different parameters differed slightly, but showed similar phase composition. It is highly likely that the hard precipitations formed in the coatings after laser remelting were represented mainly by M_12_C type carbides and, to a lesser degree, by M_6_C and M_2_C carbides along with WC residues, uniformly distributed in the metal matrix consisting of fcc γ-Ni solid solution, with Co, Cr, Fe, Si, W dissolved in it, and complex Ni-B-Si eutectic based on the Ni_3_B, Ni_3_Si_2_ and Ni_31_Si_12_ compounds.
- The hardness of laser-processed coatings ranged from ~1030 to ~1310 HV0.2, which was up to ~2 times higher than that of the coatings remelted in a furnace. The sample preheating did not cause a visible reduction in hardness. With the introduction of oscillation, the hardness dropped and leaps were observed, which may be associated with a too-low layer thickness, when a non-uniform (at the micro scale) distribution of WC particles in the layer to be remelted had a significant effect.
- After the laser remelting, the wear resistance of the coating improved by ~2.9 times and the friction coefficient reduced by ~21%, as compared with the coatings remelted in a furnace.

## 5. Conclusions

Laser remelting with laser beam oscillation is an effective method for post-processing of the surface of MMC Ni-based/WC coatings, resulting in a significant increase of the coating microhardness and improvement in wear resistance, along with the reduction of the friction coefficient.

Taking into account all the study results, it is reasonable to increase a preheating temperature to completely prevent the formation of cracks in the coating and apply a higher laser power during the post-processing to obtain deeper melting and more uniform hardness of the coating.

## Figures and Tables

**Table 1:** Parameters of laser processing and number of tests.

Oscillation Amplitude *A*, mm	Laser Operating Speed *v_x_*, mm/min			
	250	500	750	1000
0 (no oscillation)	+	+	+	+
1	+	+	+	+
2	+	+	+	+

“+”—the experiment was performed.

**Table 2:** Elemental composition of phases in furnace remelted coating (EDS; at.%).

Element	Cr-Rich Phases				WC		W,Cr-Rich		γ-Ni		Ni-B Eutectic	
	Sp.1	Sp.2	Sp.3	Sp.4	Sp.5	Sp.6	Sp.7	Sp.8	Sp.9	Sp.10	Sp.11	Sp.12
B	10.65	9.55	12.28	10.42	1.16	-	10.28	2.41	0.25	6.47	26.41	25.56
C	36.02	38.58	39.22	39.83	57.78	69.47	40.95	40.89	23.28	22.21	21.57	21.65
Si	0.02	-	-	-	-	-	3.90	4.47	2.06	2.09	-	-
Cr	45.59	45.12	38.59	41.54	-		14.20	15.74	7.30	7.15	4.77	4.00
Fe	2.34	2.17	2.19	1.33	1.06	0.83	1.30	2.97	11.29	10.75	5.52	5.23
Co	-	0.40	2.34	1.88	-	-	2.15	2.49	6.82	4.68	3.95	4.60
Ni	5.36	3.81	4.42	4.16	0.47	0.47	19.46	22.86	48.59	46.26	37.68	38.88
W	0.02	0.37	0.97	0.84	39.53	29.23	7.76	8.19	0.40	0.39	0.13	0.07
Total	100.00	100.00	100.00	100.00	100.00	100.00	100.00	100.00	100.00	100.00	100.00	100.00

**Table 3:** Elemental composition of phases in laser-remelted coatings (by EDS; at. %).

Element	Cr,W-Rich				Ni,Cr,W-Rich					γ-Ni		Ni-B Eutectic	
	Sp.1	Sp.2	Sp.9	Sp.10	Sp.3	Sp.4	Sp.11	Sp.12	Sp.13	Sp.5	Sp.6	Sp.7	Sp.8
B	8.59	6.52	0.20	3.00	5.06	8.89	7.35	1.71	13.69	5.34	3.46	18.28	17.04
C	46.47	50.65	57.80	57.78	36.35	34.77	38.78	38.19	38.66	20.80	20.88	19.32	20.14
Si	-	-	-	-	2.83	2.93	3.34	4.35	1.42	2.91	3.16	2.25	3.17
Cr	22.55	22.98	22.64	19.00	12.59	13.07	12.89	16.43	8.16	6.52	4.55	4.25	3.20
Fe	4.00	2.08	0.77	2.00	5.06	3.71	3.38	3.51	4.01	8.46	8.94	5.68	5.13
Co	2.14	0.36	0.26	0.94	3.93	3.17	3.03	3.26	3.07	6.10	6.21	5.21	5.01
Ni	2.69	1.86	1.43	1.72	26.16	25.54	21.57	23.59	19.29	48.82	51.62	44.65	46.06
W	13.55	15.55	19.94	18.39	8.03	7.93	9.67	8.96	11.71	1.06	1.18	0.36	0.24
Total	100.00	100.00	100.00	100.00	100.00	100.00	100.00	100.00	100.00	100.00	100.00	100.00	100.00

**Table 4:** Results of the tribological tests.

Sample	Friction Coefficient	Coating Mass Loss, μg	Contra-Body Mass Loss, μg	Coating Wear Rate, μg/m	Coating Wear Resistance, m/mg
As-sprayed	0.54 ± 0.039	17.22 ± 2.83	1.397 ± 0.143	86.1 ± 14.16	11.8 ± 2.0
Furnace	0.42 ± 0.016	0.663 ± 0.006	0.403 ± 0.138	3.32 ± 0.029	301.5 ± 2.61
Laser + Preheating	0.33 ± 0.028	0.233 ± 0.025	0.367 ± 0.083	1.17 ± 0.126	863.7 ± 91.7

## Data Availability

Not applicable.
